# Effectiveness and Limitations of Unsupervised Home-Based Balance Rehabilitation with Nintendo Wii in People with Multiple Sclerosis

**DOI:** 10.1155/2015/916478

**Published:** 2015-10-25

**Authors:** Massimiliano Pau, Giancarlo Coghe, Federica Corona, Bruno Leban, Maria Giovanna Marrosu, Eleonora Cocco

**Affiliations:** ^1^Department of Mechanical, Chemical and Materials Engineering, University of Cagliari, 09123 Cagliari, Italy; ^2^Multiple Sclerosis Centre, Department of Public Health, Clinical and Molecular Medicine, University of Cagliari, 09126 Cagliari, Italy

## Abstract

Balance training represents a critical part of the rehabilitation process of individuals living with multiple sclerosis (MS) since impaired postural control is a distinctive symptom of the disease. In recent years, the use of the Nintendo Wii system has become widespread among rehabilitation specialists for this purpose, but few studies have verified the effectiveness of such an approach using quantitative measures of balance. In this study, we analyzed the postural sway features of a cohort of twenty-seven individuals with MS before and after 5 weeks of unsupervised home-based balance training with the Wii system. Center of pressure (COP) time-series were recorded using a pressure platform and processed to calculate sway area, COP path length, displacements, and velocities in mediolateral (ML) and anteroposterior (AP) directions. Although the results show a significant reduction in sway area, COP displacements, and velocity, such improvements are essentially restricted to the ML direction, as the Wii platform appears to properly stimulate the postural control system in the frontal plane but not in the sagittal one. Available Wii games, although somewhat beneficial, appear not fully suitable for rehabilitation in MS owing to scarce flexibility and adaptability to MS needs and thus specific software should be developed.

## 1. Introduction

In persons living with multiple sclerosis (MS), the reduction of the physical impairments associated with the disease represents one of the main goals of the rehabilitation process [1, 2]. In particular, since people with MS often show impaired postural control with consequent increased risk of falls [3], it is important to include specific balance training routines in the rehabilitation plan.

While different approaches (strength, resistance and aerobic training, hippotherapy, kinesio taping, whole body vibration, sensory integration balance training, etc.) have been tested for this purpose with encouraging results [4–12], in recent years the use of devices originally designed for entertainment purposes has become widespread (see the recent exhaustive review by Taylor and Griffin for further details [13]). In fact, several studies report significant improvements in balance of MS patients following both fully or partly supervised training periods (as part of a rehabilitation program) performed using the Nintendo Wii console in addition to the Balance Board and Wii-Fit software [14–18]. It is believed that the positive effects observed are due to biofeedback mechanisms, activated mainly through the visual system, even though the actual modalities of stimulation of the postural control system induced by this device have been scarcely investigated.

It is noteworthy that the outcome measures employed to assess the effectiveness of the Wii training program are mixed since balance has been assessed using either clinically rated (e.g., Berg Balance Scale, Equiscale, and the Dynamic Gait Index) or patient-reported outcome measures (such as the Activities-Specific Balance Confidence Scale) or objective instrumental measurements. Thus, there are difficulties in correctly evaluating the actual impact of this approach, as well as in generalizing the results obtained.

Few studies have evaluated changes in balance due to Wii training through postural sway analysis obtained from center-of-pressure (COP) position time-series acquired by a force platform during a quiet standing test [16–18]. This technique has been proven reliable in characterizing the performance of the postural control system in a variety of conditions, which include either suppression of the visual input or alteration of proprioceptive information [19]. Moreover, in a prospective study on people with MS, it was observed that abnormal sway values (particularly as regards the COP path length) are associated with a greater risk of falls [20].

Brichetto et al. [16] analyzed the sway area (i.e., 95% confidence ellipse) in presence and absence of visual input in two groups of individuals with MS. One of them performed 12 sessions (articulated in three 60-minute sessions/week) of supervised Wii activity, while the other (matched for age and Expanded Disability Status Scale (EDSS) score) underwent conventional balance training exercises. Although significant reductions in sway area were detected in both individuals, the statistical analysis revealed that there was a greater effect over time for the Wii-group versus the control group as regards closed-eye stabilometry. Similarly, Prosperini et al. [18] found a significant reduction in the COP path length (i.e., the overall length of the trajectory followed by the COP during the quiet standing trial) in a crossover study that analyzed balance before and after a period of 12 weeks (48 30-minutes sessions) of partly supervised home-based Wii activity in 36 patients with MS divided into two groups. They also observed that in one of the groups analyzed the training effects on static balance were substantially maintained even 12 weeks after completion of training.

Apart from the limited, not homogeneous, and somewhat incomplete data on postural sway changes originated by Wii training, there are some issues related to the use of this platform that have not yet been fully clarified. For example, the results of tests to characterize the postural strategies adopted by a patient during an actual Wii rehabilitation session showed that the Wii-Fit games commonly used for this purpose induce an unbalanced activity in sagittal and frontal planes (i.e., significantly higher COP displacements and velocities in the mediolateral direction) [21]. This phenomenon is not dependent on the pathology since it is evident with similar magnitude even in healthy individuals. Moreover, while in most previous studies the patient's activity was supervised, the great advantage of the use of the Wii platform in the home environment appears to be that the balance training can be self-administered, thus reducing the costs of the whole rehabilitation plan. Nevertheless, there is no information on possibly reduced effectiveness of this approach in absence of continuous training guidance.

Thus, on the basis of the aforementioned considerations, this study intends to assess the effect of 5 weeks of unsupervised home-based balance rehabilitation performed with Nintendo Wii, Balance Board, and Wii-Fit software, by means of postural sway analysis. In particular, the set of sway parameters considered for the evaluation of treatment effectiveness will be expanded in comparison with previous investigations including COP displacements and velocities (not previously considered). The hypothesis to be tested is that the balance training performed with the Nintendo Wii has different effects in the anteroposterior (AP) and mediolateral (ML) directions.

## 2. Methods

### 2.1. Participants

**Figure 1:**
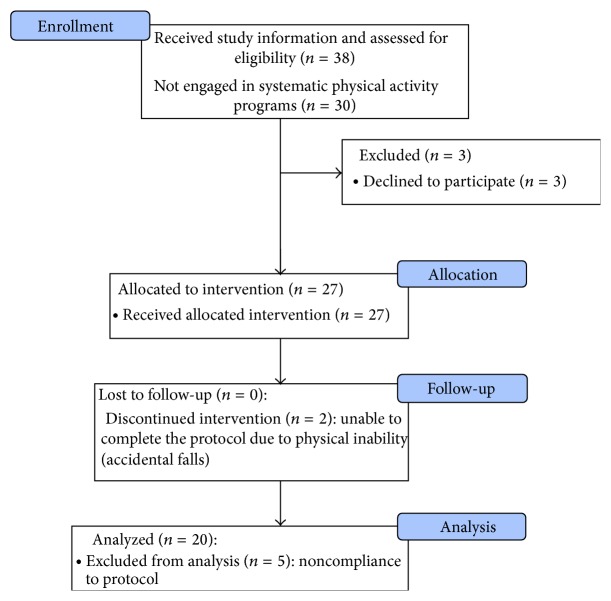
Flow of participants through the study.

In the period of January-February 2014, a convenience sample of 38 outpatients with MS followed at the Regional Multiple Sclerosis Centre of Cagliari (Sardinia, Italy) was informed about the study by the neurologists of the center and assessed. Individuals who met the following criteria were considered eligible for the study: diagnosis of MS according to the 2005 McDonald criteria [22] and the ability to sustain a stable upright posture for at least 15 minutes, which was the minimum time required for a Wii typical balance training session. Moreover, to avoid the influence of confounding factors in the assessment of the effectiveness of the Wii-assisted balance training program, patients who were already engaged in systematic physical activity programs were excluded. The screening for eligibility criteria and the Expanded Disability Status Scale (EDSS) score attribution were performed by a neurologist experienced in MS (GC, EC, and MGM). A subgroup of 30 individuals was enrolled in the study (Figure 1). Three of them declined to participate after a few days because of lack of time.

Kick-start meetings were organized and held between March and May 2014 by a team of neurologists, physiotherapists, and engineers. In that circumstance the participants (divided into three groups) were given a Nintendo Wii Mini console, a Balance Board, the Wii-Fit software suite, and a written memorandum with the schedule of the training protocol. Detailed instructions and a practical demonstration on the basic use of the platform and the type of games to play were also delivered.

The local ethics committee approved the study and all participants signed an informed consent agreeing to participate in the study.

### 2.2. Training Sessions

The training was planned over a 5-week period, with 5 compulsory sessions per week and a minimum of 30 minutes per day of exercise, dividable (at participants' discretion depending on their fatigue state) into two 15-minute sessions. Instead, no limits were imposed as regards the maximum training time: participants were allowed to use the console as much as they liked thus self-administering a possible surplus of training. In any case, the daily time spent playing was recorded in the Wii console log and subsequently analyzed to verify adherence to the protocol.

We expected participants to cumulate at least 12.5 hours of training during the period of study. This value was selected after considering all previous studies involving Wii use by people with MS, in which total training time ranged from 6 to 24 hours, with single-session durations from 15 to 60 minutes, and who reported balance improvements due to the training program [16–18]. All the games selected for the training, namely, Penguin Slide, Table Tilt, and Balance Bubble, belong to the Wii-Fit suite and are among the most commonly employed in similar studies on individuals with MS [15, 16, 18]. Participants were instructed to equally divide the training time with the three games. For safety reasons, although no participants needed mobility aids to sustain the upright posture, we allowed them to arrange the presence of supports around the Balance Board location to use if/when needed.

### 2.3. Postural Sway Measurements

Postural sway was assessed before and after the training period on the basis of the analysis of COP time-series acquired using a digital force platform (BTS P6000, BTS Bioengineering, Italy) with acquisition frequency set at 480 Hz. Participants were asked to stand barefoot as still as possible for 30 seconds on the platform, having the feet placed on two 30°-oriented footprints (intermalleolar distance of 8 cm) drawn on a paper sheet placed on the force platform, while maintaining a stable and relaxed position with the arms freely positioned by the sides and the gaze fixed on a target image placed at a distance of 3 m, adjusted to each participant's eye level. These procedures ensured a common reference position, and foot placement followed recommendations of the International Society of Posturography [23]. Tests were repeated in two conditions, namely, with eyes open and closed. Three trials for each condition were acquired, allowing a suitable rest time between them.

The raw COP time-series were low-pass filtered (10 Hz cutoff, 4th-order Butterworth, bidirectional) and then postprocessed with a custom-developed MATLAB (The MathWorks, Inc., Natick, MA, USA) routine to calculate the following parameters:

- sway area (SA, 95% confidence ellipse, mm^2^),
- COP path length (the overall distance travelled by the COP during the trial, mm),
- COP maximum displacement (the difference between the maximum and minimum values of the selected coordinate recorded during the trial, mm) in the AP and ML directions,
- COP velocity (calculated as the average of the instantaneous values recorded during the trial, mm s^−1^) in the AP and ML directions.

### 2.4. Statistical Analysis

A two-way (vision × time) repeated measures ANOVA was conducted using SPSS software (v.20, IBM, Armonk, NY, USA) to examine the effect of balance training on the aforementioned sway parameters. The level of significance was set at *p* = 0.05. When necessary, a post hoc Holm-Sidak test for pairwise comparison was carried out to assess intra- and intergroup differences. Data were preliminarily checked for normality and equal variance using the Shapiro-Wilk and Levene tests.

## 3. Results

Of the 30 patients assessed for eligibility to enter the study, 3 declined to participate; thus 27 people started the balance training program. Two persons dropped out due to injuries consequent to accidental falls (not related to the use of the Nintendo Wii) and thus data of 25 individuals were available. Moreover, the analysis of the activity logs recorded by the Wii console revealed that 5 other participants did not fully comply with the predefined schedule (e.g., they skipped some sessions or trained less than 30 minutes per day) and thus they were excluded from the analysis. These subjects were interviewed after the log analysis and when requested to explain the poor compliance, they generically reported “lack of time” and “scheduling problems” as the main reasons for the incomplete training.

Descriptive statistics for the 20 patients who correctly and completely followed the training program are reported in Table 1.

The values of the selected postural sway parameters measured before and after the training protocol are summarized in Table 2.

ANOVA revealed main effect of training as regards sway area (*F*_(1,19)_ = 10.96,  *p* = 0.004, and Wilks *λ* = 0.63) and COP displacements and velocity in the ML direction (*F*_(1,19)_ = 12.11, *p* = 0.003, and Wilks *λ* = 0.61) for displacements and (*F*_(1,19)_ = 4.83, *p* = 0.041, and Wilks *λ* = 0.79) for velocity.

Main effects of vision were also observed for all the sway parameters: sway area (*F*_(1,19)_ = 13.27, *p* = 0.002, and Wilks *λ* = 0.59), COP path length (*F*_(1,19)_ = 28.25, *p* < 0.001, and Wilks *λ* = 0.40), COP displacements in ML direction (*F*_(1,19)_ = 11.65, *p* = 0.003, and Wilks *λ* = 0.61), COP displacements in AP direction (*F*_(1,19)_ = 52.29, *p* < 0.001, and Wilks *λ* = 0.27), COP velocity in ML direction (*F*_(1,19)_ = 17.37, *p* < 0.001, and Wilks *λ* = 0.52), and COP velocity in AP direction (*F*_(1,19)_ = 35.27, *p* < 0.001, and Wilks *λ* = 0.35).

In particular, the post hoc analysis revealed that the sway area decreased by 19% only for the eyes open condition (*p* = 0.006), the COP displacements in ML direction decreased in both visual conditions (eyes open: −11%, *p* = 0.049; eyes closed: −18%, *p* < 0.001), and COP velocity in ML direction significantly decreased only in presence of visual input (−10%, *p* = 0.048). No significant time-per-vision interactions were found.

**Figure 2:**
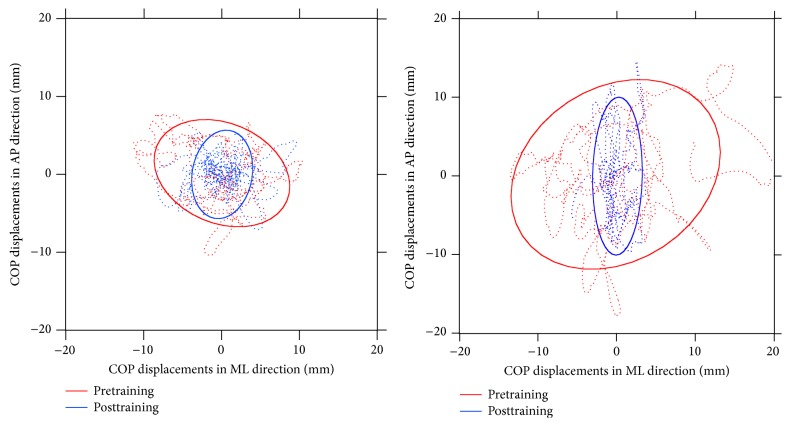
Example of sway path and areas acquired before and after Wii training.

Some examples of the sway paths and areas calculated before and after the Wii training are shown in Figure 2.

## 4. Discussion

Our purpose was to characterize postural sway changes in individuals with MS following 5 weeks of unsupervised home-based balance rehabilitation performed using the Nintendo Wii system.

Although several previous studies reported the usefulness of such an approach in improving balance abilities in individuals with MS, few of them specifically employed objective quantitative techniques (i.e., use of force platforms) to assess the quantitative changes in postural sway associated with the balance training [16–18].

In particular, our results are consistent with those of Guidi et al. [17] and Brichetto et al. [16] who found a reduction in sway area after 10 and 12 hours of Wii training. In particular, Brichetto et al. [16], similar to what was observed here, reported sway area reduction in presence and absence of visual input, although more markedly with respect to the present investigations (40% versus 20%). On the contrary, we found no significant improvements in COP path length as reported by Prosperini et al. [18] who observed a reduction in this parameter in the range of 15–17%, even though the baseline values of the COP path length were similar, thus indicating that, despite the differences in equipment, such COP measurements are very robust and reliable for balance assessment in individuals with MS. Such discrepancies are probably due either to the different duration/intensity of the training or to the existence of supervision by a therapist. As regards the latter aspect, several studies in different fields of neuro- and physical rehabilitation observed that although in both supervised and unsupervised exercise programs benefits for the patients were observed, supervised programs are associated with greater improvements in the investigated function [24, 25]. Also to be noted is that some differences may have been introduced by the foot position on the platform during the posturographic tests, as this variable is able to influence the sway values [26].

It is noteworthy that some improvements in postural control performances (i.e., reduction of COP displacements in ML direction) were found even when participants were tested in absence of visual input, despite the fact that the training was mainly based on visual feedback. This phenomenon was observed in similar previous studies [27, 28] and explained with the fact that the use of visual feedback during training is probably able to induce some kind of recalibration process of the sensory inputs, thus globally ameliorating the effectiveness of the postural control system.

The main novelty of the present study is represented by a more refined analysis of sway parameters that includes assessment of COP maximum displacements and velocities subdivided by anatomical plane. Our data show that the improvements associated with Wii training are restricted to the ML direction. This would indicate that the commercially available software commonly used to train individuals with MS (i.e., the Wii-Fit suite) differentially stimulates postural control activity in the sagittal and frontal planes.

Previous experiments performed to characterize the postural strategies of individuals with MS during actual rehabilitation sessions [21] showed that COP displacements and velocities in the ML direction were significantly higher with respect to the AP direction. Moreover, such a phenomenon is present even in the case of healthy individuals, and thus such unbalanced activity is likely due to the structure of the Wii-Fit games, which mainly require subjects to shift their weight from one limb to the other while reduced movements are required in the AP direction.

Indeed, the focal clinical point of our results is as follows: are we sure that we are saying that the Wii balance system leads to the best performance achievable? In fact, even though a “global” beneficial effect is introduced by the Wii training in people with MS (expressed through sway area or COP path length reduction), the Wii-Fit exercises lead to unbalanced activity in AP and ML directions in terms of COP displacements and velocities. This is likely due to the fact that this system is designed for healthy people and recreational purposes and thus may not be fully suitable for achieving controlled improvement in postural control performances.

One possible way to overcome such difficulties would be the use of dedicated software that exploits Balance Board capabilities as the input device and is also designed to meet the specific training requirements and allows therapists to define the type, duration, and difficulty of exercises depending on patients' needs and degree of impairment. Such an approach was used, for example, by Young et al. [29] and Gil-Gómez et al. [30] who built and validated customized games to train balance in older adults and individuals affected by brain injury. It is noteworthy that in the study by Young et al. [29] similar reductions of sway after training were observed in both the ML and AP directions, thus indicating that balance training with Nintendo Wii was able to stimulate postural control in a well-balanced manner.

Some limitations of the study are to be acknowledged: firstly, our design study did not include a control group, and this fact limits the possibility of attributing the changes in postural sway we observed exclusively to the effect of Wii training. Secondly, we tested only three games of the Wii-Fit suite, selecting those most widely used in previous studies on people with MS. Further studies with larger samples and a wider variety of games would be desirable to see if the unbalanced postural activity in ML direction detected here is actually to be associated with intrinsic features of the Wii system. Finally, it is still unclear whether the effect of a therapist's supervision may or may not mitigate (or remove completely) possible confusing factors affecting the results due to incorrect performance of the training which is possible in the home setting, as well as reducing the number of noncompliant participants.

## 5. Conclusion

The results of the present study confirm that 5 weeks of unsupervised home-based balance rehabilitation training performed using the Nintendo Wii with Balance Board and Wii-Fit games significantly reduced several postural sway parameters of individuals living with MS, thus indicating an improvement in the performance of the postural control system.

Nevertheless, there are some issues associated with such an approach that suggest caution in its use or, at the very least, further in-depth analyses. In particular, balance training appears to be much more intense in the ML than in the AP direction: in fact, the significant changes observed here as regards COP displacements and velocities involve in practice only the frontal plane. Moreover, the scarce flexibility of the Wii-Fit software in terms of exercise difficulty and scoring system make it difficult for physiotherapists to administer and customize training in accordance with the patient's initial conditions and improvements. Thus, also considering the possibilities of exploiting the interesting features of the Balance Board as a training tool even when not used in conjunction with a Wii console (as it can be connected to a common Personal Computer), future studies should address the development and testing of software specifically designed for MS needs.

## Figures and Tables

**Table 1:** Baseline demographics and clinical characteristics of the participants.

Variable	Mean values	Range
Age (years)	44.6 ± 10.6	18.3–60.2
Body mass (kg)	62.6 ± 10.4	46.0–88.0
Stature (cm)	166.2 ± 7.7	150–178
BMI (kg m^−2^)	22.9 ± 3.8	18.6–31.2
EDSS score	3.4 ± 1.3	1.5–6.0
Total Wii training time (h)	24.23 ± 12.82	12.6–52.9

**Table 2:** Variation of postural sway parameters before and after the Wii training for bipedal stance in eyes-open and eyes-closed conditions.

	Eyes open			Eyes closed			Time effect *p* value	Vision effect *p* value	Time × vision interaction *p* value
	Pre-Wii	Post-Wii	Pre-post change	Pre-Wii	Post-Wii	Pre-post change			
Sway area (mm^2^)	340.68 ± 197.82	275.85 ± 195.73	−64.83 ± 75.62	502.08 ± 337.78	389.44 ± 339.54	−97.96 ± 199.44	0.004^†^	0.002^†^	0.482
COP path length (mm)	469.85 ± 142.91	463.52 ± 163.92	−6.33 ± 84.54	700.95 ± 308.83	668.12 ± 330.90	−26.52 ± 159.63	0.502	<0.001^†^	0.521
COP max disp. ML (mm)	25.12 ± 9.54	22.36 ± 9.00	−2.76 ± 5.43	31.82 ± 13.66	25.98 ± 11.15	−5.13 ± 6.67	0.003^†^	0.003^†^	0.131
COP max disp. AP (mm)	29.38 ± 6.68	29.18 ± 10.08	−0.21 ± 6.66	39.02 ± 9.67	37.26 ± 13.98	−1.30 ± 11.47	0.629	<0.001^†^	0.704
COP velocity ML (mm s^−1^)	8.94 ± 2.89	8.07 ± 3.20	−0.86 ± 1.83	12.29 ± 5.60	11.09 ± 5.78	−0.96 ± 2.72	0.041^†^	<0.001^†^	0.932
COP velocity AP (mm s^−1^)	10.78 ± 3.86	8.11 ± 3.20	−2.16 ± 5.19	17.02 ± 7.87	16.72 ± 8.77	−0.25 ± 4.77	0.892	<0.001^†^	0.507

All values are expressed as mean ± SD. Pre-Wii: baseline values, Post-Wii: after 5 weeks of balance training with Nintendo Wii. The symbol ^†^denotes statistical significance (*p* < 0.05).
